# Effects of Emotional Expressiveness of a Female Digital Human on Loneliness, Stress, Perceived Support, and Closeness Across Genders: Randomized Controlled Trial

**DOI:** 10.2196/30624

**Published:** 2021-11-25

**Authors:** Kate Loveys, Mark Sagar, Xueyuan Zhang, Gregory Fricchione, Elizabeth Broadbent

**Affiliations:** 1 Department of Psychological Medicine The University of Auckland Auckland New Zealand; 2 Soul Machines Ltd Auckland New Zealand; 3 Auckland Bioengineering Institute The University of Auckland Auckland New Zealand; 4 Department of Psychiatry Harvard Medical School Boston, MA United States; 5 Benson-Henry Institute of Mind Body Medicine Massachusetts General Hospital Boston, MA United States

**Keywords:** computer agent, digital human, emotional expressiveness, loneliness, closeness, social support, stress, human-computer interaction, voice, face, physiology

## Abstract

**Background:**

Loneliness is a growing public health problem that has been exacerbated in vulnerable groups during the COVID-19 pandemic. Social support interventions have been shown to reduce loneliness, including when delivered through technology. Digital humans are a new type of computer agent that show promise as supportive peers in health care. For digital humans to be effective and engaging support persons, it is important that they develop closeness with people. Closeness can be increased by emotional expressiveness, particularly in female relationships. However, it is unknown whether emotional expressiveness improves relationships with digital humans and affects physiological responses.

**Objective:**

The aim of this study is to investigate whether emotional expression by a digital human can affect psychological and physiological outcomes and whether the effects are moderated by the user’s gender.

**Methods:**

A community sample of 198 adults (101 women, 95 men, and 2 gender-diverse individuals) was block-randomized by gender to complete a 15-minute self-disclosure conversation with a female digital human in 1 of 6 conditions. In these conditions, the digital human varied in modality richness and emotional expression on the face and in the voice (emotional, neutral, or no face; emotional or neutral voice). Perceived loneliness, closeness, social support, caring perceptions, and stress were measured after each interaction. Heart rate, skin temperature, and electrodermal activity were assessed during each interaction. 3-way factorial analyses of variance with post hoc tests were conducted.

**Results:**

Emotional expression in the voice was associated with greater perceptions of caring and physiological arousal during the interaction, and unexpectedly, with lower feelings of support. User gender moderated the effect of emotional expressiveness on several outcomes. For women, an emotional voice was associated with increased closeness, social support, and caring perceptions, whereas for men, a neutral voice increased these outcomes. For women, interacting with a neutral face was associated with lower loneliness and subjective stress compared with no face. Interacting with no face (ie, a voice-only black screen) resulted in lower loneliness and subjective stress for men, compared with a neutral or emotional face. No significant results were found for heart rate or skin temperature. However, average electrodermal activity was significantly higher for men while interacting with an emotional voice.

**Conclusions:**

Emotional expressiveness in a female digital human has different effects on loneliness, social, and physiological outcomes for men and women. The results inform the design of digital human support persons and have theoretical implications. Further research is needed to evaluate how more pronounced emotional facial expressions in a digital human might affect the results.

**Trial Registration:**

Australia New Zealand Clinical Trials Registry (ANZCTR) ACTRN12621000865819; https://www.anzctr.org.au/Trial/Registration/TrialReview.aspx?id=381816&isReview

## Introduction

### Background

Loneliness is a growing public health issue that has been exacerbated in vulnerable groups during the COVID-19 pandemic [1]. It is a subjective psychological state in which a person perceives a discrepancy between the quality and quantity of their actual and ideal social relations [2]. It has been described as a *syndrome-like* condition that reliably affects social cognition and behavior in a manner that perpetuates feelings of loneliness [3-5]. Loneliness has been shown to be most prevalent in young adults (aged 18-29 years); older adults; people with a physical or mental health condition; people with low income; people who live alone; and people who are separated, widowed, or divorced [1,6].

Loneliness is a particularly pressing public health problem because it has been associated with a multitude of ill health effects, which places a burden on health care systems [3,7]. Loneliness produces a greater mortality risk than smoking 15 cigarettes every day [8], and it increases the risk of many physical and mental morbidities. This includes a greater risk of coronary heart disease, stroke [9], and psychiatric conditions, including major depressive disorder, generalized anxiety disorder, and suicide [10,11]. Interventions are needed to alleviate the burden of loneliness on population health and health care systems.

### Social Support Interventions

Social support has been shown to be a major protective factor against loneliness in adults [6,12] and an effective intervention strategy for loneliness [4]. In a survey of 38,217 adults in the United Kingdom, a high level of social support was associated with 89% lower odds of developing severe loneliness [12]. Moreover, reporting ≥3 close relationships was associated with 42% lower odds of severe loneliness. This suggests that interventions for improving social support and close relationships may help combat loneliness, which has been found in a systematic review of loneliness interventions [4].

Social support is a construct that refers to the structure or function of interpersonal relationships [13]. Structural social support describes aspects of a person’s social network, including the existence of relationships (eg, number of friends) or the types of connections between people (eg, spousal). Functional social support describes the functions of a particular relationship or interaction for a person (eg, emotional, informational, instrumental, or appraisal support). Both structural and functional support have been associated with reductions in loneliness to varying degrees [5]. However, satisfaction with functional support that has been received has been found to have the strongest relationship with loneliness. This suggests that social support interventions focused on improving functional support could have the strongest effect on loneliness.

Psychological interventions have been shown to increase social support [14]. This may be through one of several techniques including teaching skills to help people strengthen their support networks or to better solicit functional support, or the direct provision of functional support to people (eg, emotional support from a support group). Psychological interventions for improving social support have been shown to reduce loneliness [4] and improve health outcomes [14]. Social support may improve health outcomes indirectly through the provision of functional support (eg, transportation to a health appointment) or directly by exerting a stress-buffering effect [15,16]. Social support has been associated with reduced sympathetic nervous system activation [17] and reduced cortisol and increased oxytocin release [18], which may help to buffer against the effects of loneliness-related stress on the body. Chronic stress has been associated with reduced heart rate variability [19], increased inflammation [17,20], and impaired immune response [21], which increases the risk of physical and mental health conditions.

Social support may have stress-buffering and loneliness-reducing effects because humans have evolved to survive in groups. Owing to this evolution, humans experience separation distress and find relief in their social attachments [22]. Social support is an *attachment solution* that is encouraged in mammalian brain structure and neural processing loops (eg, the *protolimbic* and *paralimbic cortico-striato-thalamocortical* loops) to provide solace and alleviate distress. Attachment solutions may be found in other people, animals, religious figures, and transitional objects (eg, a child’s teddy bear) [22]. In-person social support interventions may not always be available (eg, because of physical restrictions during a pandemic, hospitalization, and living rurally). When faced with the absence of human connection, artificial agents (such as robots or computer agents) might provide digital social support to people that may be salutogenic in the context of loneliness.

### Robot and Computer Agent Support

Research has increasingly shown that social robots and computer agents may help to reduce loneliness and improve health outcomes for people by providing functional social support. A social robot is a technology with a body in physical hardware form, which is programmed to perceive and act autonomously within its physical environment and is capable of social interaction [23]. For example, Paro is a fluffy baby harp seal robot that includes artificial intelligence for social interactions and a heater for body warmth. Paro may provide emotional support to people through companionship and physical touch akin to a pet. Paro has been shown to reduce loneliness in older adults, and reduce pulse rate and blood pressure [24-26]. Other robots have reduced loneliness by facilitating interactions between people and their support networks over video calls [27,28].

A computer agent refers to a computer-generated entity that may include a dialog system and an embodiment (eg, an animation of a face or body) [29,30]. Computer agents are often capable of social interaction and may include technologies such as embodied conversational agents, chatbots, game characters, and digital humans. There is limited research investigating the effect of support from a computer agent on loneliness [31]. However, 1 study found that companionship from an animal-like conversational agent reduced loneliness in hospitalized older adults [32]. Other studies have found that Tanya, a humanlike computer agent companion for older adults at home, was effective at improving loneliness and was highly acceptable to users [33,34]. Similarly, studies in young adult populations have found that computer agents show promise for improving loneliness [35,36].

Digital humans are a new form of computer agent that show promise for applications in health care, including acting as a supportive peer. Digital humans are computer-based, autonomous animations in the form of a human face or a full body. They use complex neurobehavioral modeling techniques involving virtual neurotransmitters and a visual computing framework described by Sagar et al [37]. These techniques enable emotional intelligence, personality, and complex social interactions. For example, digital humans can show attachment and separation distress toward a user while in *high oxytocin mode*. *High oxytocin mode* is a setting that involves more rapid firing of virtual oxytocin in the digital human’s autonomous brain model when a human is detected in front of the computer’s webcam [38]. This setting influences the digital human’s emotional expressions to become increasingly distressed when the user goes out of view and become more positive when the user returns. As digital humans exist on a screen, they may be better suited to providing emotional and informational support to people as opposed to instrumental support (eg, helping with physical tasks).

Digital humans (and computer agents in general) are a new technology, and research is needed to understand how to design them in a way that is conducive to providing support to people. Research has shown that the perceived effectiveness of social support interventions may be influenced by how socially close people feel to the support partner [39]. This suggests that it may be important to design digital humans to engage in behaviors that build closeness with people while providing support. However, there is limited research on which behaviors increase closeness with computer agents [40].

### Improving Relationships With Computer Agents

Psychological research has suggested that closeness can be developed in human relationships using several techniques. These include engaging in reciprocal self-disclosure, undertaking shared activities or interests, expressing the value of the relationship, and showing empathy, among others [41,42]. However, there may be gender differences in the techniques that are most important for building closeness [41,43,44]. Women may place greater importance on emotional self-disclosure in close relationships, whereas men may find shared ideas and hobbies to be more important to closeness [41,44]. It is unclear whether behavioral techniques that increase closeness in human relationships translate to human and computer agent relationships; however, the computers are social actors (CASA) paradigm would suggest so [45].

CASA posits that people interact with computers that provide social cues in a manner similar to how they would with another person [46]. Findings from several experiments support CASA [45], showing that people make personality judgments [47] and gender stereotypes [48], engage in mindless social behaviors such as reciprocity [45], and elicit in-group and outgroup behaviors toward computers [49]. More recently, studies have shown that CASA applies to advanced technologies. A voice agent in an autonomous vehicle was perceived more positively when its communication style conformed to gender stereotypes (eg, a sociable female voice vs an informative male voice) [50]. Another study found that the similarity-attraction principle applied to a voice agent in a smart-home environment [51]. Extroverted users were found to prefer a talkative voice agent, whereas introverted users preferred an agent with multiple voices because it felt like talking to several less-talkative agents. According to CASA, computer agents that display closeness-building behaviors should form closer relationships with people than agents that do not.

### Emotional Expressiveness

Emotional expressiveness is a technique that can increase closeness in human relationships, particularly in female relationships [41,44]. Emotional expressiveness refers to expressing an intimate degree of negative or positive affect (eg, through facial expression, speech, and gaze), usually while engaging in behaviors such as talking at a personal level about fears or personal problems, or while demonstrating care [44]. Emotional expressiveness may help build emotional intimacy, a type of psychological intimacy that is important in close relationships [52]. It may also be a way of providing nurturance, which is one of the 3 types of social attachment–building behaviors in mammals, according to the Mammalian Behavioural Triad theory [53]. As digital humans have a detailed animated face modeled on human musculature and a voice, they are capable of engaging in multimodal emotional expression. This involves delivering congruent emotional cues through different communication modalities, such as facial expressions and speech tone.

Although promising, research has yet to investigate whether emotional expressiveness in a digital human increases feelings of closeness toward it. Related research on social robots has found gender differences in the effect of emotional expressiveness on closeness. Women have been shown to feel closer to a social robot that expresses emotions on the face during interactions (alongside other relational behaviors, eg, self-disclosure, mirrored posture, and speaking rate), whereas men reported feeling closer to robots that expressed fewer emotions and relational behaviors [54]. It is possible that similar effects could be found with computer agents; however, experimental research is needed to investigate this.

Prior research on computer agents has shown that emotional expressiveness can increase social perceptions related to closeness. A computer agent that expressed empathy on the face and in the voice was rated as significantly more supportive, caring, and likable compared with an agent that displayed more self-focused emotion (eg, joy at its own wins in a game) [55]. Similarly, another computer agent that displayed a range of emotions on the face and in the voice was perceived as significantly more warm, caring, cooperative, and trustworthy than an agent with no emotion [56].

These studies show that emotional expressiveness in a computer agent may increase perceptions related to closeness; however, no studies have looked at the effects on closeness directly. In addition, from the existing research, it is unclear whether emotions expressed through the face or voice are more important for closeness building and supportive interactions with a computer agent. Some studies have shown that the presence of a face may help to increase attentional engagement with a computer agent [57], which could improve how well users attend to emotional cues. However, it remains unclear whether the presence of a computer agent face can affect the development of social closeness. Several instruments have been developed to quantify closeness, including the Inclusion of Other in the Self scale [58], the Perceived Interpersonal Closeness Scale [59], and the Relationship Closeness Induction Task (RCIT) measure (for experimentally induced feelings of closeness) [60].

### This Study

Research has yet to investigate the effects of emotional expressiveness in a digital human’s face and voice on user outcomes, and how emotional expressiveness interacts with user gender during a supportive interaction. The aim of this study is to investigate the effect of emotional expressiveness in a female digital human on loneliness, closeness, caring perceptions, social support, stress, and physiological arousal in a community sample. It was hypothesized that there would be gender differences in the effect of emotional expressiveness in a female digital human on outcomes. Particularly, it was anticipated that women would report the greatest reductions in loneliness, stress, and physiological arousal and the greatest increases in social support, closeness, and caring perceptions in response to a female digital human with an emotional face and an emotional voice. In contrast, it was predicted that men would report better outcomes in response to a female digital human with a neutral face and a neutral voice.

## Methods

### Study Design

A between-group experimental study was conducted to investigate the effect of multimodal emotional expression and user gender on loneliness after a self-disclosure interaction with a female digital human. Secondary outcomes included social closeness, caring perceptions, social support, stress, and physiological arousal. Participants were block-randomized by gender to one of 6 conditions in which the digital human’s design differed by face type (emotional, neutral, or no face) and voice type (emotional or neutral; described in the *Digital Human* section).

The study procedures were approved by the University of Auckland Human Participants Ethics Committee on November 1, 2018 (reference number: 022191). Retrospective registration was provided by the Australia New Zealand Clinical Trials Registry (registration number: ACTRN12621000865819). Retrospective registration was sought as the study was conceived of as a human-computer interaction experiment (to understand the effect of a digital human’s design) as opposed to a clinical trial. However, later registration was sought, given the number of psychological and physiological outcomes collected as part of the experiment. This study is reported in keeping with the CONSORT (Consolidated Standards of Reporting Trials) 2010 guidelines [61].

### Participant Recruitment

A community sample of 198 adults (101 women, 95 men, and 2 gender-diverse individuals) was recruited to participate in a study on relationships with a digital human. Participants were considered eligible if they were aged ≥18 years and were fluent in English. The study was advertised using flyers distributed in the University of Auckland campuses and through Facebook advertising to the Auckland city area. Compensation of a NZ $20 (US $14.33) shopping voucher was offered for participation. Participants were recruited between February 20, 2019, and July 24, 2019.

A power analysis was conducted using G*Power software to determine the required sample size. This was informed by the results of Brave et al [55], who compared the effects of agent empathy versus no empathy on feelings of support and found an effect size of *f*=0.32. 198 participants would be needed to detect an effect size of *f*=0.32, with 80% power and an α level of .05 for a 3 (face) by 2 (voice) by 2 (gender) analysis of variance (ANOVA). A recruitment target was set for 100 women and 100 men.

**Figure 1:**
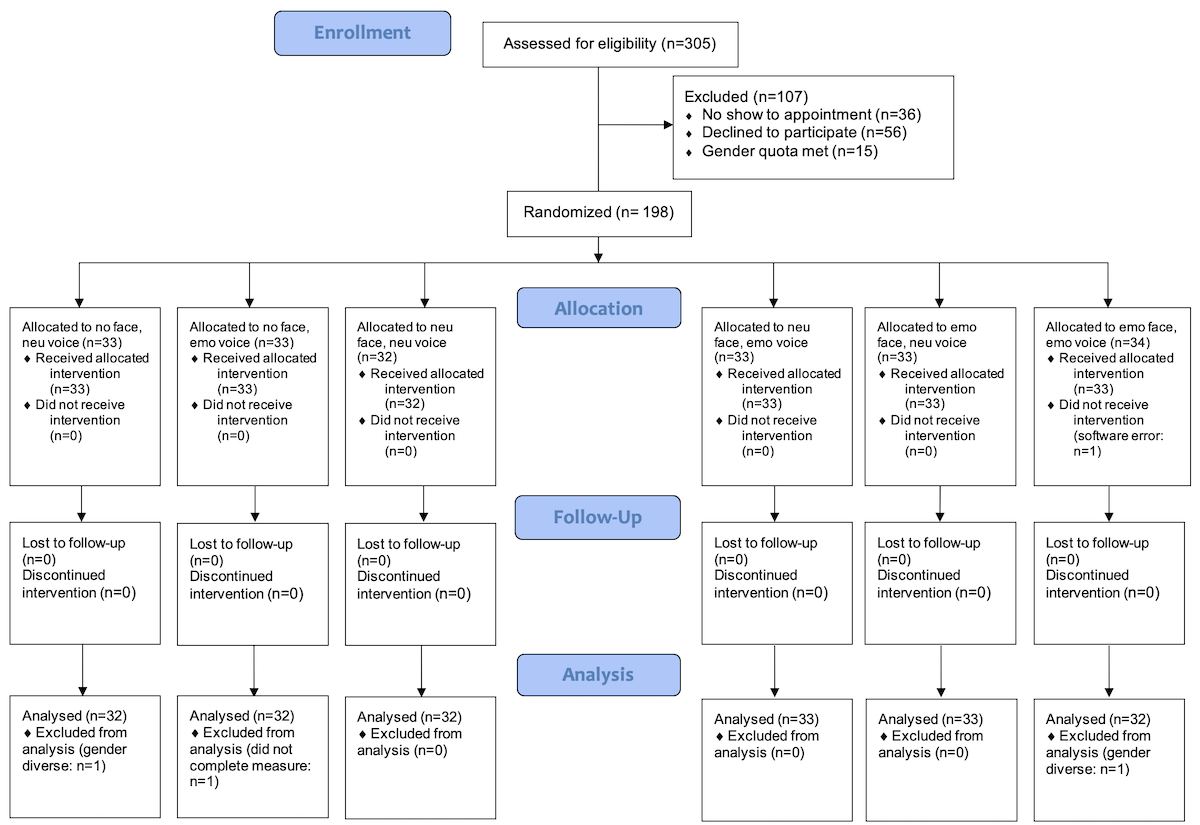
CONSORT (Consolidated Standards of Reporting Trials) flow diagram (emo: emotional; neu: neutral).

Data from 1.5% (3/198) of participants were excluded from analyses for the following reasons: the software would not start (1/198, 0.5%) or the participant was identified as gender diverse, and this group was not of sufficient size for adequate statistical power in gender analyses (2/198, 1%). As a result, data from 195 participants were included in the analyses. A CONSORT diagram depicts participant flow through the study (Figure 1).

### Measures

**Figure 2:**
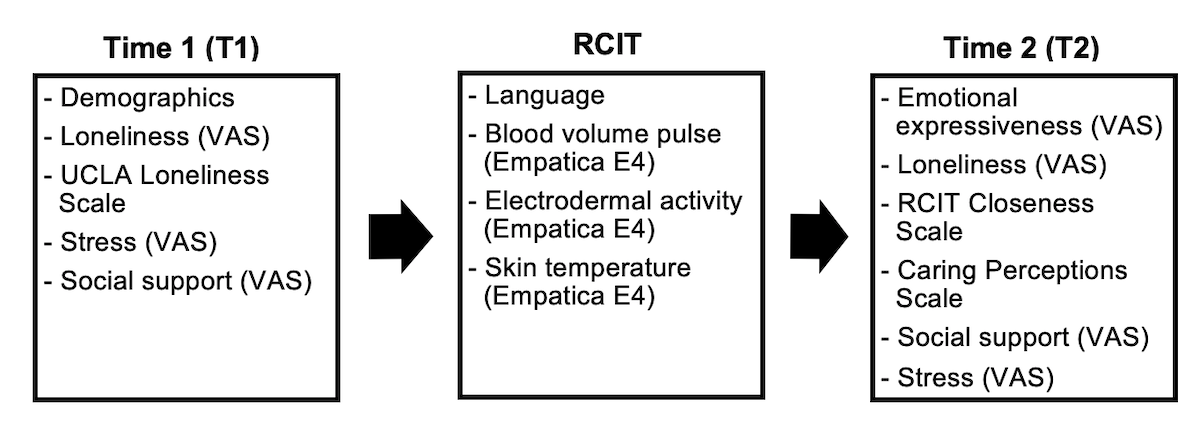
Time points for administration of measures and data collection. RCIT: Relationship Closeness Induction Task; VAS: visual analog scale.

Figure 2 depicts the time points at which the measures were administered and data were collected in the study.

#### Demographics

Demographic variables including age, gender, ethnicity, marital status, and occupation were assessed by self-report at baseline. Gender was assessed using a single-response, multiple-choice question with response options (male, female, gender-diverse, and prefer not to divulge). Other demographic variables were evaluated using an open-ended self-report item (age) and multiple-choice questions with response options (ethnicity, marital status, and occupation).

#### Perceived Emotional Expressiveness Manipulation Check

A manipulation check evaluated the extent to which the digital human was perceived as emotionally expressive using a visual analogue scale. Participants marked their answers by placing an X at the appropriate position along a 100-mm line with response anchors at either end (0=*not expressive*; 100=*very expressive*). A score was derived by measuring the distance of the X along the line from the left-hand side. A higher score indicated that the digital human was perceived as more emotionally expressive. Perceived emotional expressiveness was measured immediately after the digital human interaction.

#### Loneliness

General loneliness was measured at baseline using the 20-item Revised UCLA Loneliness Scale [62]. Participants were asked to rate how often they felt the way described in general across a range of statements using a 4-point Likert scale (1=*never*; 4=*often*). The Revised UCLA Loneliness Scale has been shown to have good internal consistency reliability (Cronbach α=.94), discriminant validity with mood measures, and concurrent validity with measures of social isolation (eg, feelings of isolation, time spent alone per day, and number of close friends) [62]. Responses were summed to derive a total loneliness score. A higher score indicated greater loneliness.

State loneliness was evaluated using a visual analogue scale with response anchors placed at each end of the scale (0=*not at all*; 100=*extremely*). Participants marked how lonely they currently felt by placing an X at the appropriate place along a 100-mm line. Scores were derived by measuring the distance of the X along the line. A higher score indicated that the participant experienced a greater degree of loneliness. A visual analogue scale was chosen as it would be more sensitive to changes in feelings of loneliness within a 1-hour experiment session compared with a general loneliness measure.

#### Social Closeness

Social closeness with the digital human was assessed using the RCIT follow-up measure [60]. This 4-item scale evaluates the degree of perceived relationship closeness, similarity, liking, and likelihood of a future friendship using a 9-point scale. Responses range from 0 (*not at all*) to 9 (*very*). Scores were derived by measuring the distance of an X placed along a 100 mm line and transforming this value to a score from 0 to 9. Scores were summed for the 4 items. A higher score indicated stronger social closeness to the conversation partner. The scale demonstrated good internal consistency reliability in this study (Cronbach α=.86).

#### Perceived Social Support

Perceived social support was measured using a visual analogue scale with response anchors (0=*not at all*; 100=*extremely*) [63]. Participants rated their extent of agreement with the statement “I feel supported” by placing an X at the appropriate spot along a 100-mm line. The distance of the X along the line was measured, and a score was derived. Higher scores indicated feeling more supported. A visual analogue scale was chosen as it may be more sensitive to changes in perceived support after a short experiment session than a general measure. Although lower in internal reliability compared with a multidimensional scale, the 1-item visual analogue scale helped to reduce participant burden and was thought to be suitable for assessing feelings of support after a brief interaction.

#### Caring Perceptions

Caring perceptions are judgments about an agent’s traits (whereas feeling supported pertains to the effect of the interaction on the user). Caring perceptions were assessed using the Caring Perceptions Scale by Brave et al [55]. This is a 5-item scale that evaluates the extent to which a computer agent is perceived as caring. Items are rated on a 10-point scale with semantic differential response anchors (eg, *not compassionate* to *compassionate*). Items in the scale evaluated how warm, compassionate, selfish, friendly, and cooperative the digital human was. This scale has been used in prior research looking at relationships with embodied conversational agents and has shown good internal consistency reliability in university student samples (Cronbach α=.88) [55]. Items were summed, and a higher score indicated that the participant perceived the digital human as more caring.

#### Perceived Stress

Current stress was measured using a 100-mm visual analogue scale with response anchors (1=*not at all*; 100=*extremely*) [64,65]. Participants rated their extent of agreement with the phrase *“I feel stressed”* by placing an X at the appropriate point along the line. The distance from the left edge of the line was measured, and a score was derived. A higher score indicated greater perceived stress. This visual analogue scale was chosen because it was shown to be sensitive to changes in perceived stress after a brief experimental session in previous research [64,65].

#### Physiological Stress Response

Heart rate, electrodermal activity, and skin temperature are measures of physiological arousal that can indicate stress [66,67]. Heart rate, electrodermal activity, and skin temperature were measured using a wrist-worn sensor device called Empatica E4 (Empatica Inc). Data were continuously collected during the interaction and subsequently processed using a Python script to create average heart rate (converted from blood volume pulse data), electrodermal activity, and skin temperature scores per participant. Higher average scores in heart rate and electrodermal activity and lower scores in wrist skin temperature indicated greater physiological arousal during the interaction with the digital human. Data were analyzed from the beginning to the end of the participant’s interaction with the digital human. Data from only 170 participants were included in the analyses for physiological stress, as 25 data files were lost to processing errors.

### Procedure

Participants attended a 45-minute experimental session at the University of Auckland Clinical Research Centre. After providing written informed consent, participants secured an Empatica E4 sensor to their wrist and completed a baseline questionnaire on paper. The baseline questionnaire assessed demographic and psychological variables, including loneliness, stress, and social support. Participants were then block-randomized by gender to interact with 1 of 6 versions of a digital human. Block randomization was completed before the session by a member of the research team, who automatically generated a randomization table using Research Randomizer software. Allocations were concealed from the researcher and the participant in opaque envelopes. The researcher remained blinded to the participant’s condition until the envelope was opened immediately before starting the appropriate computer program for the participant. Although the participants were deblinded to their condition upon starting their interaction, they remained unaware of what digital humans in the other experimental conditions were like. The 6 digital humans varied in terms of modality richness and emotional expression (emotional face, neutral face, no face; emotional voice, and no voice).

The researcher provided the participant with verbal and written instructions for interacting with the digital human, started the digital human program on the laptop computer, then closed the door, and exited the room for the duration of the interaction. Participants were instructed to call on the researcher (who was sitting on a chair down the hallway) by ringing a loud desk bell if they encountered any technical difficulties that they could not resolve.

Participants completed the RCIT with a digital human. The RCIT is a 15-minute structured conversation involving reciprocal self-disclosure of personal information, feelings, and memories over a list of 28 personal questions [60]. Questions gradually increase in intimacy and cover topics from “What is your name?” to “Describe the last time you felt lonely.” The RCIT has been shown to reliably induce a sense of closeness among human-stranger dyads in experimental psychology research [60], and it has been shown to promote health benefits found in naturally occurring close relationships, including faster wound healing speed and reduced stress [64]. The RCIT is divided into 3 sections with a time limit for each (2, 5, and 8 minutes). To maintain a private interaction between the participant and the digital human, the participant was required to ring a loud desk bell each time they reached the end of a section. The researcher sat in the hallway running a digital timer that was cleared at the beginning of each section. If a participant went over the time limit for a given section, the researcher knocked on the door and the participant was instructed to move on to the next section. Participants’ language from their interaction with the digital human was recorded and analyzed for emotional content in another paper [68]. Once the RCIT was completed, the participant rang the desk bell, and the researcher returned to the room. The participant removed the wrist sensor and completed a second paper questionnaire on perceived loneliness, closeness, caring perceptions, social support, and stress. Participants also answered a series of open-ended, written qualitative questions on their perceptions of the digital human (reported in another paper; Loveys, unpublished data, March 2021). Once the questionnaire was completed, the participant was provided with a NZ $20 (US $14.33) shopping voucher.

### Digital Human

#### Overview

**Figure 3:**
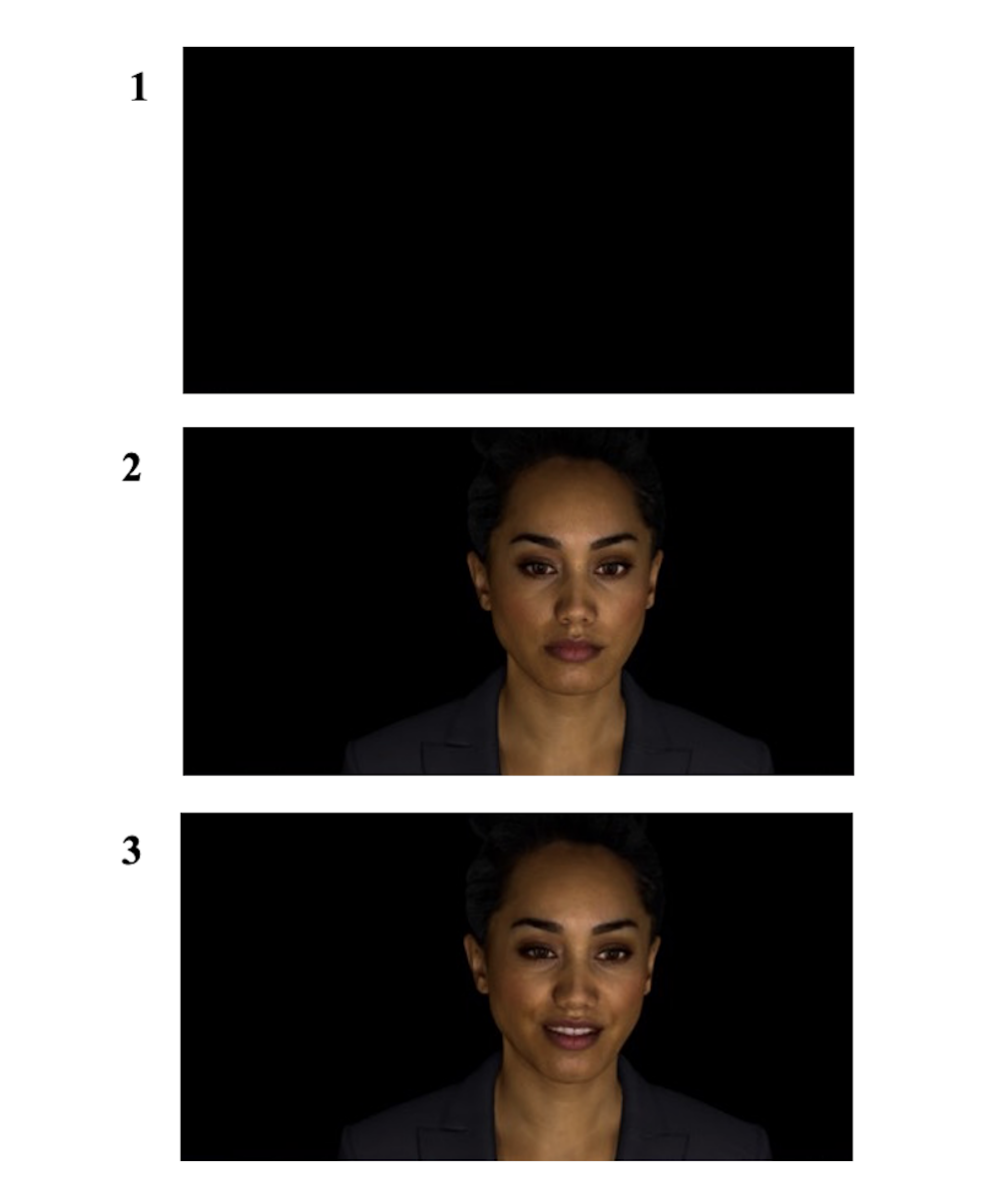
The digital human across the 3 visual conditions (1: no face; 2: neutral face; 3: emotional face). Each visual condition could be paired with either an emotional or neutral voice.

The digital human was presented on a laptop computer screen in a portrait view with an embodiment of the head and shoulders depicted in front of a black background. The digital human was named Holly, and she was modeled on a young adult female of mixed ethnicity (New Zealand Māori, New Zealand European, Indian, and European). In the *no-face* conditions, the digital human appeared as a black screen only. Figure 3 illustrates how the digital human appeared in the no-face, neutral face, and emotional face conditions. The digital human functioned autonomously using a finite-state conversation engine and a neurobehavioral modeling and visual computing framework, as described by Sagar et al [37].

Before programing the digital human’s emotional expressions, a member of the research team (KL) recruited 4 psychology master’s students to complete the RCIT in 2 dyads (1 female-female; 1 male-male). Each dyad completed the task separately in a private room with the researcher present. The researcher recorded notes on the types of emotions expressed by the dyads during each of the 28 questions in the conversation task. Notes were also made regarding the types of responses people gave to different questions. This information was used to inform the answers the digital human gave to the RCIT questions and the types of emotions it expressed on the face and in the voice for each question. This approach was taken to improve how natural the digital human’s responses were. The digital human’s language was consistent between participants, as were the emotional expressions on the face and in the voice for individuals in those respective conditions. This design choice was made to maintain experimental control against the potential confounds of different conversation content or amounts of emotional expression, which could have affected the outcomes.

The digital human’s script underwent an iterative development process involving the research team drafting responses to the RCIT questions, programming responses into the digital human as part of a finite-state conversation engine, and then testing the conversation with other members of the research team. The digital human’s responses were refined over several iterations to make them seem natural and convincing. The digital human interacted autonomously with participants.

#### Emotional Face

The emotional face condition included a mixture of positive and negative expressions, including compassion, joy, fear, and sadness. Compassion and joy were expressed most frequently (113/213, 53% and 82/213, 38.5% of the interactions, respectively). The emotional expressions were subtle in order to seem more natural. The emotional expressions on the face were preprogrammed and triggered based on the phrases that the digital human spoke using a text-to-speech Emotional Markup Language. Programing the Emotional Markup Language required manually classifying each sentence the digital human spoke into compassion, joy, fear, or sadness categories in the digital human program. The result was that each time the digital human spoke a phrase, she elicited the associated emotional expression on her face. Emotional expressions on the face were autonomously generated in real time as the digital human spoke to the participant using neurobehavioral modeling and visual computing techniques. These techniques are described in detail by Sagar et al [37,38]. While listening, the digital human did not express emotions on the face, although she engaged in humanlike facial movements, such as blinking, as described in the *Other Behaviors* section.

The neutral face condition contained the same digital human face as the emotional face condition; however, no facial expressions were made during the interaction. The only movements the neutral face made were of the mouth to accommodate speech and the natural behaviors described in the *Other Behaviors* section (eg, blinking and head movements with speech). In the no-face condition, participants interacted with a plain black screen that contained no digital human animation and only a voice.

The digital human was capable of mirroring people’s facial expressions; however, this capability was turned off for the experiment. This was to ensure that the digital human did not provide emotional facial expressions in the neutral face condition and that participants in the emotional face condition received the same amount of emotional expression from the digital human. However, the tradeoff was that the digital human could not return a person’s smile, for example.

#### Emotional Voice

Participants interacted with a digital human that had either an emotional or neutral voice. The digital human’s voice was recorded by a young adult female voiceover artist with a local New Zealand accent. Emotions expressed in the emotional voice condition were compassion (ie, low arousal positive affect; 113/213 statements, 53% of the conversation), joy (ie, high arousal positive affect; 82/213 statements, 38.5% of the conversation), and sadness or anxiety (ie, low arousal negative affect; 18/213 statements, 8.5% of the conversation). The neutral voice had an absence of positive and negative emotions; however, it maintained normal speech intonation for a New Zealand accent. Each sentence in the digital human’s script was recorded twice—one in a neutral voice and the other in an emotional voice. For the emotional voice, the voiceover artist read from a script with annotations to guide when certain emotional expressions should take place. These emotional expressions were tied to phrases to correspond with when emotions were portrayed in the digital human’s face. Recording the voice clips was an iterative process where a member of the research team (KL) and an audio engineer provided subjective feedback to the vocal artist at the time of recording, and the vocal artist rerecorded clips where necessary. Clips were rerecorded when the emotional expression was either too strong or not strong enough or where there appeared to be emotion in a neutral voice clip.

To ensure that the emotional and neutral voices were sufficiently different, an emotion classifier was used to objectively analyze the vocal clips. The classifier operated with 79.9% accuracy and classified vocal clips based on energy and pitch contours, which were modeled using Gaussian mixture models. Analyses revealed that the compassionate and joyful emotional voice clips were significantly different from the neutral voice clips. However, there were insufficient sadness and anxiety voice clips to reach significance. The voice recordings were generated in a voice package that was connected to a finite-state conversation engine.

#### Other Behaviors

The digital human engaged in several other behaviors to appear more humanlike during the interaction. She maintained eye contact with people for the majority of the interaction using computer vision technology. Before speaking, she would often look at the top-right of the screen as if thinking. She also engaged in humanlike movements, such as blinking, moving her head, raising her eyebrows with speech, and lightly swaying her body while listening or speaking. If she did not understand the participant, she would say, “I didn’t understand, you can try rephrasing.” The digital human did not perform any other behaviors during the interaction.

### Data Analysis

Data were analyzed using SPSS software, version 27 (IBM Corporation). Data were checked for violations of test assumptions, and bootstrapping was applied to tests where data were not normally distributed. Chi-square tests and ANOVA tests were conducted to check for baseline group differences in demographic and psychological variables. As no significant differences were found in the baseline variables, they were not controlled for in subsequent analyses. A series of 3-way factorial ANOVA tests were conducted to evaluate the effect of face type, voice type, and gender on outcomes at time point 2 (T2). Post hoc tests with Sidak correction were applied as follow-up analyses.

## Results

### Participants

Participants were predominantly young adults (mean 28.31, SD 10.97), single (96/195, 49.2%), and university students (132/195, 67.7%). There was an approximately equal number of men (94/195, 48.2%) and women (101/195, 51.8%). Participants represented a mix of ethnicities (80/195, 41.0% Asian; 67/195, 34.4% New Zealand European; 38/195, 19.5% other; 6/195, 3.1% Māori; and 4/195, 2.1% Pacific Peoples). Participants reported a moderate degree of loneliness at baseline (mean 36.97, SD 10.04). There were no significant differences in baseline demographic or psychological variables between the experimental groups, and there were no significant differences in psychological variables between men and women at baseline.

### Manipulation Check

A manipulation check revealed that participants perceived the emotional face and voice conditions as more emotionally expressive. There were significant main effects of face type (*F*_2,183_=6.97; *P*=.001) and voice type (*F*_1,183_=3.94; *P*=.049) on perceived emotional expressiveness of the digital human. An emotional face was rated as significantly more emotionally expressive than a neutral face (mean 53.02, SD 27.33, vs mean 40.14, SD 23.06; *P*=.01). No face was rated as more emotionally expressive than a neutral face (mean 55.17, SD 24.90; *P*=.002). In terms of voice type, emotional voice was rated as significantly more emotionally expressive than neutral voice (mean 52.79, SD 24.73, vs mean 45.91, SD 26.71; *P*=.049). There were no other significant main, 2-way, or 3-way interaction effects (all *F* values <1.60).

At times, errors with the conversation engine meant that the digital human occasionally interrupted the participants during their speech. However, interruptions were equally likely across the experimental conditions (*F*_5,179_=1.11; *P*=.36).

### Perceived Loneliness (T2)

**Figure 4:**
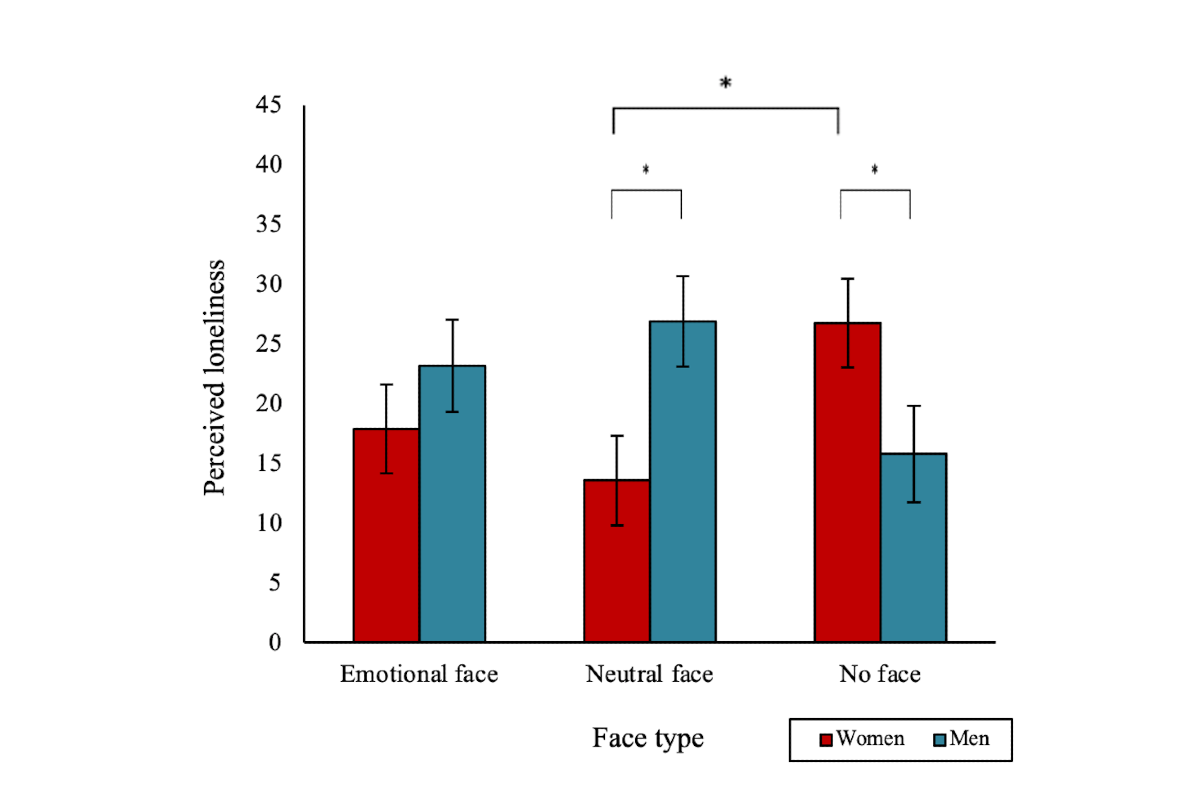
A significant two-way interaction effect of face type and gender on perceived loneliness after the digital human conversation (*P<.05). Bars indicate the SE.

Analyses revealed gender differences in the effect of a digital human’s face type on perceived loneliness. There was a significant 2-way interaction effect between face type and user gender (*F*_2,182_=4.95; *P*=.008; Figure 4). A digital human with no face was associated with significantly less loneliness in men than in women (mean 15.76, SD 19.07, vs mean 26.71, SD 24.86; *P*=.047). A neutral face digital human was associated with significantly less loneliness in women than in men (mean 13.55, SD 17.11, vs mean 26.88, SD 26.64; *P*=.01). A neutral face digital human was also associated with significantly less loneliness in women than a no-face digital human (*P*=.04). There were no other significant main, 2-way, or 3-way interaction effects (all *F* values <1.68).

### Social Closeness

**Figure 5:**
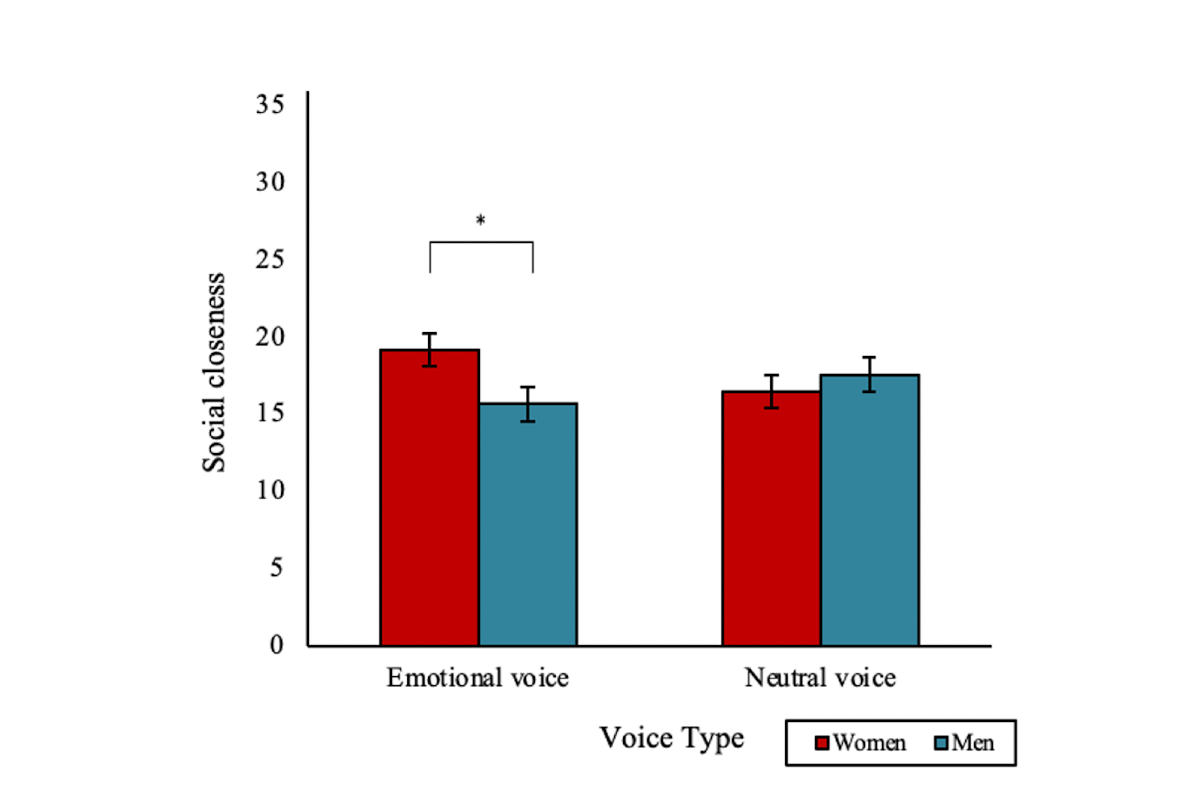
A significant two-way interaction effect of voice type and gender on social closeness with a digital human after the conversation (*P<.05). Bars indicate the SE.

An emotional voice increased feelings of closeness toward a digital human but only in women. There was a significant 2-way interaction effect between voice type and gender (*F*_1,181_=4.17; *P*=.04; Figure 5). Women reported significantly greater closeness with an emotional voice digital human than men did (mean 19.18, SD 7.21, vs mean 15.68, SD 7.53; *P*=.02). There was a trend toward women reporting greater closeness with an emotional voice digital human compared with a neutral voice digital human (mean 16.45, SD 7.06; *P*=.07). There were no other significant main, 2-way, or 3-way interaction effects (all *F* values <1.06).

### Perceived Support (T2)

**Figure 6:**
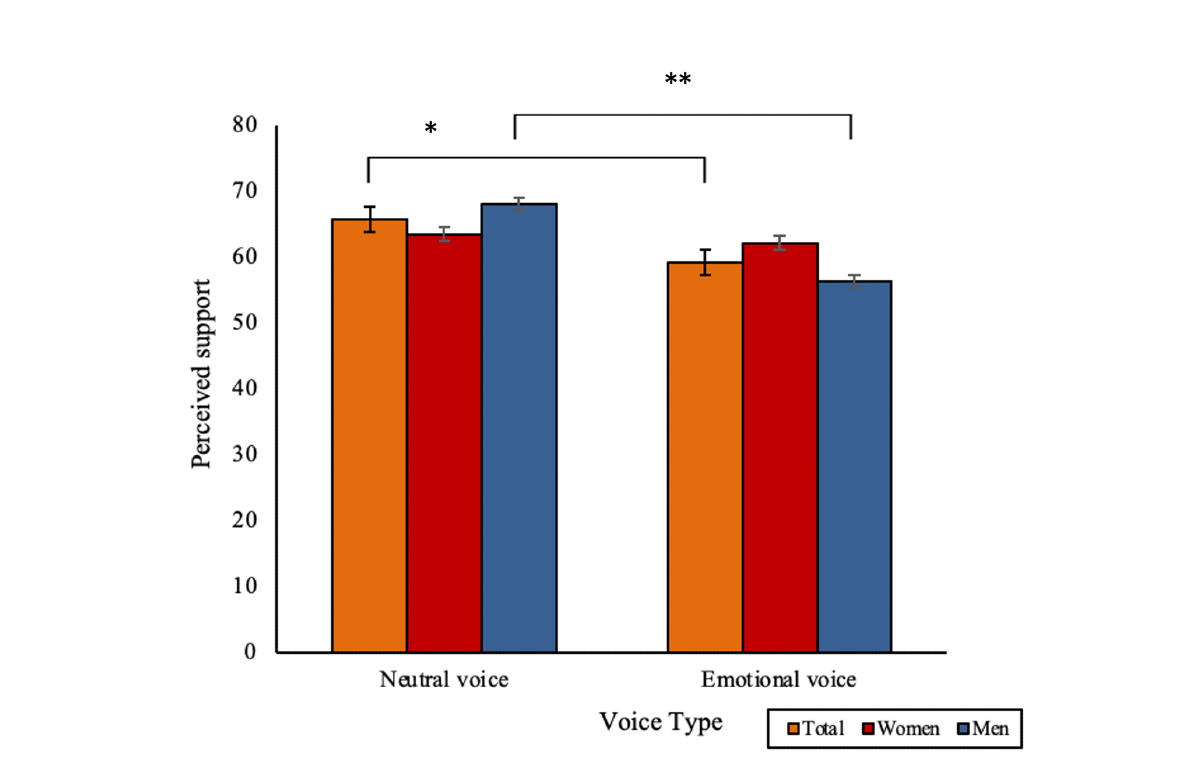
A significant main effect of digital human voice type on perceived support after the digital human conversation (*P<.05 and **P<.01). Bars indicate the SE.

A neutral voice was associated with significantly greater feelings of support across men and women. Analyses revealed a significant main effect of voice type on perceived support (*F*_1,182_=5.77; *P*=.02). A neutral voice digital human was associated with significantly higher ratings of perceived support than an emotional voice digital human (mean 65.77, SD 19.09, vs mean 59.23, SD 19.01; *P*=.02; Figure 6).

Analyses also revealed that there may be gender differences in how supported people felt in response to emotional and neutral voices in digital humans. There was a trend toward a significant 2-way interaction effect between voice type and gender (*F*_1,182_=3.66; *P*=.06). Men felt significantly more supported with a neutral voice digital human than with one with an emotional voice (mean 68.03, SD 18.49, vs mean 56.29, SD 18.38; *P*=.002), whereas women felt more supported with an emotional voice digital human than men (mean 62.18, SD 19.25, vs mean 56.29, SD 18.38), however, this was a trend toward significance (*P*=.10). There were no other significant main, 2-way, or 3-way interaction effects (all *F* values <2.33).

### Caring Perceptions

An emotional voice was found to increase caring perceptions in both men and women. Analyses revealed a significant main effect of voice type on caring perceptions (*F*_1,183_=4.26; *P*=.04). An emotional voice digital human was rated as significantly more caring than a neutral voice digital human (mean 29.45, SD 6.56, vs mean 27.42, SD 6.94; *P*=.04).

**Figure 7:**
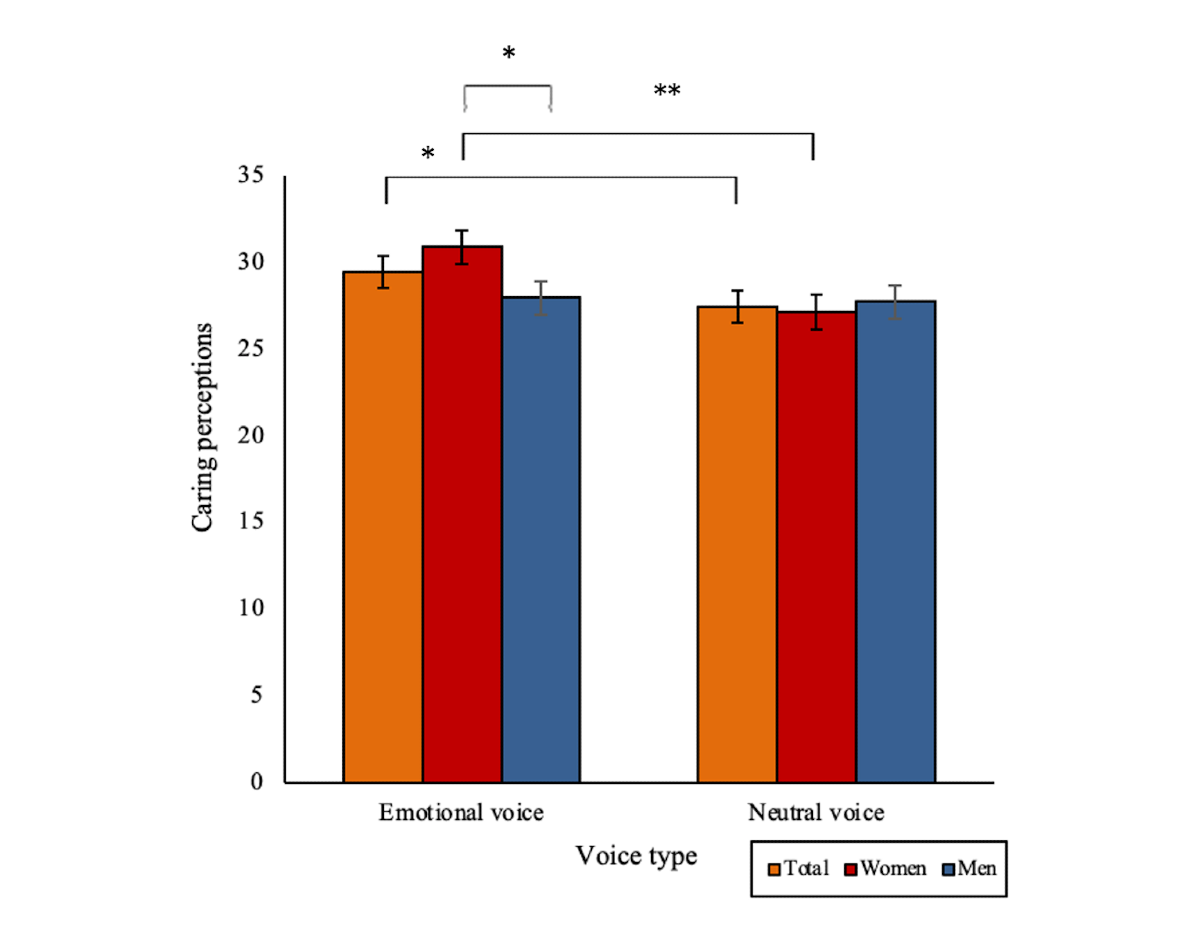
A significant main effect of voice type on caring perceptions, with a trend for an interaction with gender (*P<.05 and **P<.01). Bars indicate the SE.

There was a trend toward a significant 2-way interaction effect between voice type and gender (*F*_1,183_=3.31; *P*=.07; Figure 7). Women rated the emotional voice digital human as significantly more caring than men did (mean 30.88, SD 5.82, vs mean 27.96, SD 6.99; *P*=.03). Women also rated the emotional voice digital human as significantly more caring than a neutral voice digital human (mean 27.14, SD 6.83; *P*=.006). There were no other significant main, 2-way, or 3-way interaction effects (all *F* values <1.55).

### Perceived Stress (T2)

**Figure 8:**
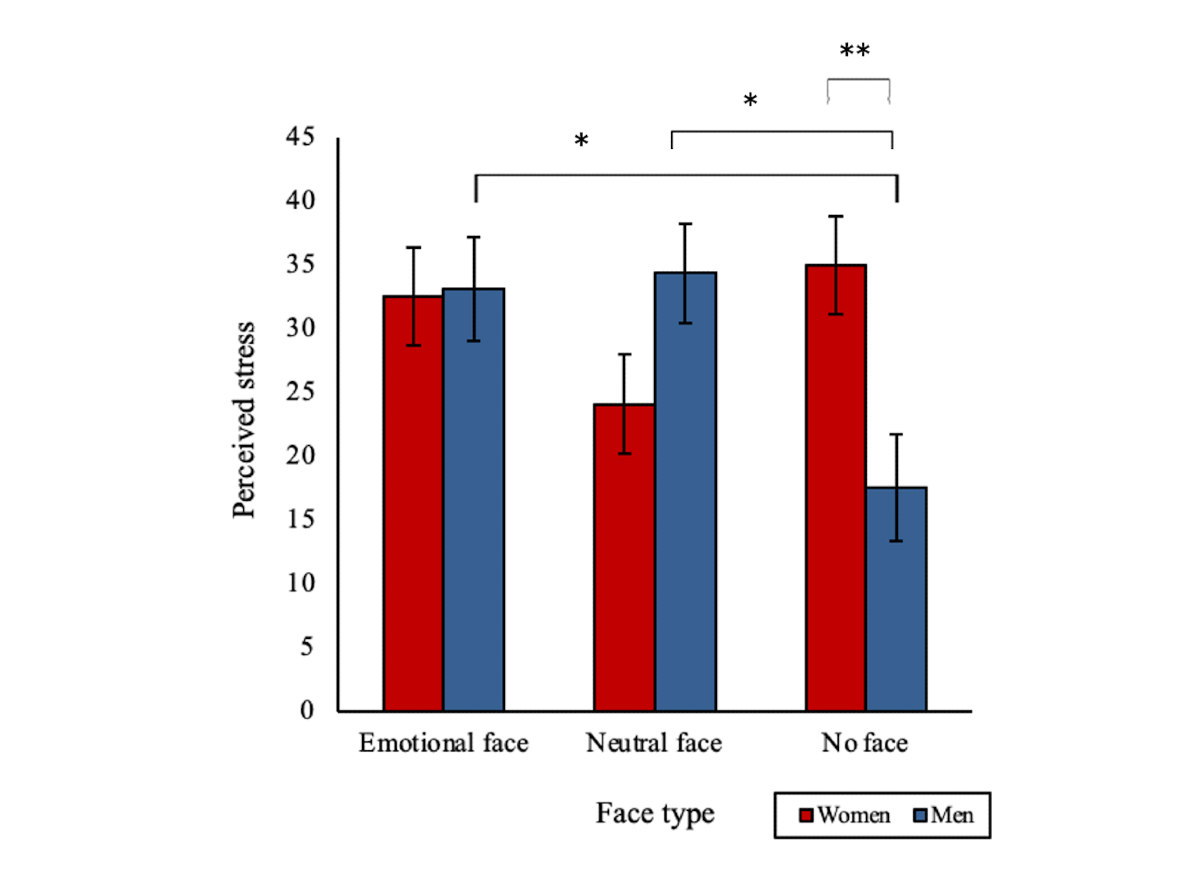
A significant two-way interaction effect of face type and gender on perceived stress after the digital human interaction (*P<.05 and **P<.01). Bars indicate the SE.

Gender was found to impact the effect of a digital human’s face type on perceived stress. A significant 2-way interaction effect was found between face type and gender (*F*_2,182_=6.01, *P*=.003; Figure 8). A nonface digital human was associated with significantly lower perceived stress in men than a neutral face (mean 17.55, SD 15.87, vs mean 34.36, SD 22.53; *P*=.01) and an emotional face digital human (mean 33.13, SD 19.09; *P*=.02), whereas women reported significantly greater stress with a no-face digital human than men (mean 35.00, SD 23.90; *P*=.003). Women also reported lower stress with a neutral face digital human than men; however, this was a trend toward significance (mean 24.09, SD 23.67; *P*=.07). There were no other significant main, 2-way, or 3-way interaction effects (all *F* values <2.68).

### Physiological Outcomes

**Figure 9:**
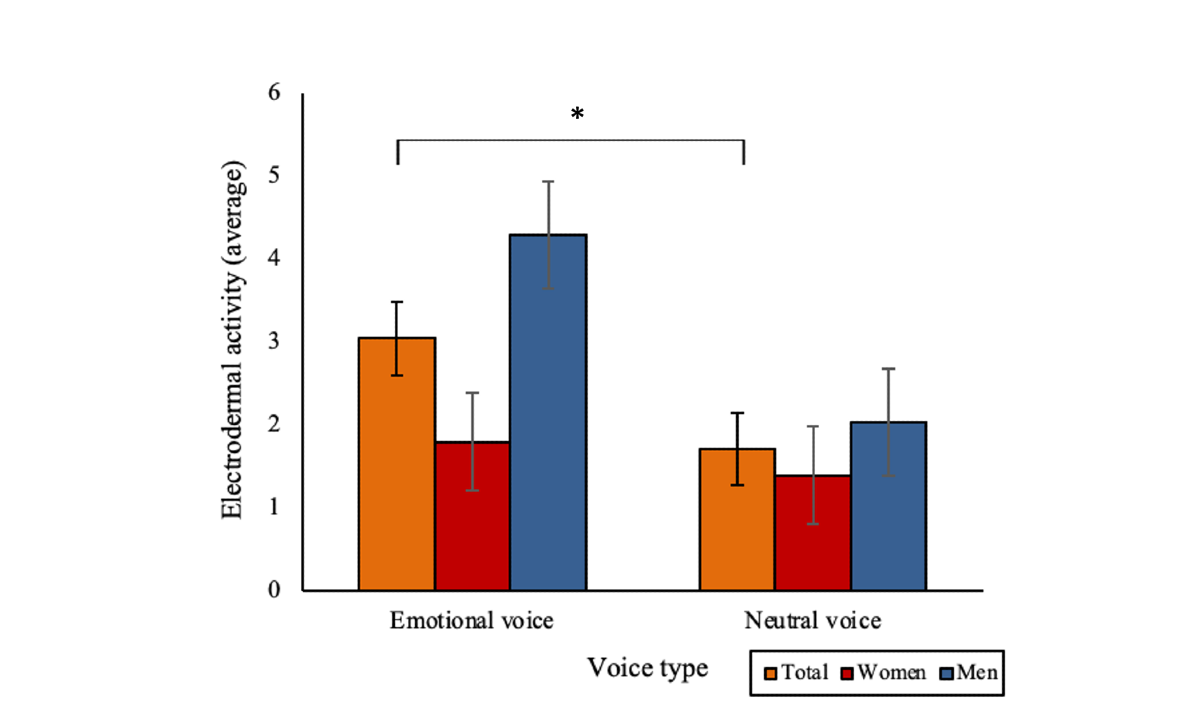
Significant main effects of voice type and gender on electrodermal activity during the digital human interaction (*P<.05). Bars indicate the SE.

There were no significant main, 2-way, or 3-way interaction effects of face type, voice type, or gender on average skin temperature (all *F* values <0.89) or heart rate (all *F* values <0.98) over the conversation. However, there were significant main effects of voice type (*F*_1,158_=4.72; *P*=.03) and gender (*F*_1,158_=6.54; *P*=.01) on average electrodermal activity. An emotional voice was associated with significantly greater electrodermal activity over the conversation than a neutral voice (mean 3.07, SD 5.38, vs mean 1.71, SD 2.13; *P*=.03; Figure 9). In addition, men experienced higher electrodermal activity during the interaction than women (mean 3.19, SD 5.69, vs mean 1.59, SD 2.04; *P*=.01). There were no other significant main, 2-way, or 3-way interaction effects (all *F* values <2.38).

## Discussion

### Principal Findings

This is the first study to investigate whether emotional expressiveness in a female digital human and user gender interact to affect loneliness, social, and physiological outcomes following a self-disclosure conversation.

Several main effects were found for the impact of emotional expressiveness in a female digital human’s voice on physiological arousal and social outcomes. Perceived social support was significantly higher for participants after interacting with a neutral voice digital human than after interacting with an emotional voice digital human. However, an emotional voice digital human was perceived as more caring overall than one with a neutral voice. Average electrodermal activity was higher during interactions with an emotional voice digital human than with a neutral voice one. These findings suggest that emotional expression in a digital human’s voice can increase physiological arousal and perceptions of caring yet reduce feelings of being supported across both genders.

Gender *did* impact the effect of emotional expressiveness on loneliness, physiological arousal, and social outcomes. Women benefited more from a female digital human with emotional expressions in the voice and with a face. For women, emotional expression in the voice increased closeness with the digital human, caring perceptions, and perceived social support. In addition, for women, the presence of a neutral face was associated with reduced loneliness and subjective stress than a digital human with no face.

In contrast, men had better outcomes with a female digital human without emotional expression in the voice and with no face (ie, a black screen). For men, a neutral voice digital human was associated with increased closeness to the digital human, caring perceptions, and perceived social support. A digital human without a face (ie, a black screen) was associated with less loneliness and lower subjective stress in men.

The potential reasons for these effects and how these findings relate to other research are discussed in subsequent sections.

### Main Effects

Emotional expression in the voice increased physiological arousal and perceptions of care yet reduced perceptions of support. The reason for why people felt less supported may be because the emotions were focused on the digital humans’ own words, as opposed to varying in response to the emotions expressed by the user. It is likely that the users expected the digital human to respond emotionally to their personal stories. Indeed, prior research has found that a computer agent that consistently delivered other focused emotions (eg, compassion) in response to users’ behavior in a game was perceived as more caring and was associated with greater feelings of support than a computer agent that delivered self-focused emotion (eg, joy at its own success in the game) [55].

The emotional voice was associated with greater physiological arousal than a neutral voice. High electrodermal activity could indicate either positively or negatively valanced arousal (eg, excitement or fear) [69]. This result is consistent with previous research showing that electrodermal activity increases when listening to emotionally valanced sounds both pleasant and unpleasant, although the greatest increases occur in response to pleasant sounds [69].

Men were found to have greater electrodermal activity than women during the interaction with the digital human. This may be explained by prior research showing that men have different brain responses to expressed emotions than women, along with different responses to viewing a female face [70,71]. For example, men and women have been shown to differentially activate brain regions in response to observing contempt and disgust facial expressions (eg, the medial frontal gyrus) [69]. This differential activation of brain regions may have downstream physical effects on the body, influencing outcomes such as electrodermal activity.

### Gender Differences

The finding that women felt closer to a digital human with an emotional voice but men felt closer to a digital human with a neutral voice is in keeping with prior literature. Emotional expressiveness is an important part of close female relationships [41,43,44], but it is a less important part of close male relationships. For men, shared activities, interests, and ideas are more important aspects of close relationships [41,44].

Similar results have been found in research examining the effect of emotional expressiveness on closeness with other types of artificial agents, including robots. Women felt closer to social robots that expressed emotions during interactions, whereas men felt closer to robots that expressed fewer emotions [54]. In research with computer agents, affective support in language was associated with greater rapport in girls, whereas for boys, an agent with only task-oriented language was associated with higher rapport [72]. The results of this study build upon the literature by showing that emotional expressiveness by computer agents can foster closer relationships for women, especially when emotions are expressed in the voice.

The gendered effects of the emotionality of the voice on perceived closeness may have also had an impact on perceived support. Women felt more supported by the emotional voice and men felt more supported by the neutral voice. This aligns with previous research showing that perceptions of social support are related to perceived closeness toward the support partner in human relationships [39].

This research also found gender effects on the impact of the digital human’s face type on perceived loneliness and stress. Men felt least lonely and stressed after interacting with a no-face digital human. In contrast, women felt the least lonely and stressed with a neutral face digital human. These results were unexpected; we predicted that women would experience the least amount of loneliness and stress with an emotional face and voice digital human. This was based on the existing literature showing lower loneliness and stress after interactions with emotionally expressive computer agents [73,74]. However, the effects of emotional expression on the face and in the voice were not compared in these studies.

This is the first study to investigate the effects of emotional expression in the voice and on the face separately in computer agents. It is possible that the effects of emotional expression are stronger when expressed in the voice than on the face. This is supported by findings that have shown that voice-only communication can increase perceptions of empathic accuracy [42]. However, as the emotional faces were very subtle in their expressions and the neutral face included humanlike movements that could have inadvertently portrayed interest (eg, raising eyebrows with speech), it is possible that the emotional and neutral face conclusions are both more about the presence of a face. This study leaves open the question of the degree of emotional expression; it is possible that different results may have been achieved with more pronounced emotional expressions on the face.

It is unclear why a neutral face was found to be more helpful for loneliness and stress in women than an emotional face, given that a manipulation check revealed that the emotional face was perceived as more emotionally expressive. It is possible that either the emotional expressions were not strong enough or the types of emotions that the digital human expressed in this study influenced outcomes. The digital human expressed a combination of self- (eg, joy and sadness) and other focused (eg, compassion and concern) facial emotions during the interaction. An interaction involving only other focused emotions could have more accurately mimicked the role of a supportive peer and had stronger effects on loneliness and stress.

Other research has shown that smiling is associated with better perceptions of a health care robot than not smiling [75]. In this study, the emotional face digital human did not smile all the time, and instead had a slight frown when expressing concern and looked sad when expressing its own sad experiences. The presence of one or both of these negative emotions might have negatively affected outcomes.

This study adds to the literature by showing that emotional expressiveness (a behavior that is important in the development of close relationships in women but not men) shows similar effects in relationships with a computer agent. In addition, the results of this study show that loneliness, stress, and social outcomes that have been associated with close human relationships can also be influenced by conversations with a computer agent. The results provide some supportive evidence for the CASA paradigm [46], which suggests that people engage in the same social behaviors toward computers that elicit social cues as they do toward other people. It makes sense that behaviors that help to build closeness in human relationships (eg, emotional expressiveness) have similar effects in relationships with a computer agent. However, technological limitations in responding may have affected cues being entirely the same as for real human conversations, as discussed in the *Limitations* section.

### Design Recommendations

Overall, the results of this study provide several design recommendations for digital humans intended for the role of a supportive peer. First, when designing a supportive peer for female users, incorporating an emotional voice may be important for promoting a closer relationship and feelings of support. For women, including an animated face was also important for reducing loneliness and perceived stress.

In contrast, when designing a supportive peer for men, a neutral voice may help to promote a closer relationship and increase feelings of support. For men, no face (ie, a black screen with a voice) was associated with less loneliness and stress than a female face with some or no emotional expression. However, it is possible that different results may have been found for men had they interacted with a male digital human.

It is important to note that these recommendations were based on results obtained with a predominantly young adult, New Zealand student sample and with a young female digital human. However, previous research has also shown gender differences in the effects of different closeness-building behaviors with other age groups in different geographic locations [41,43,44].

These findings may not generalize to all cultures. It is possible that people’s preferences regarding different aspects of emotional expressiveness (eg, type and strength) may shift at the intersection of gender and other demographic characteristics, such as culture. Prior research has shown cultural differences in targets for emotional expressiveness, including how emotions are expressed and what types are the most socially desirable to portray [76,77]. For example, in East Asian cultures, it is more socially desirable to express low arousal positive affect (eg, calm), and negative emotions are less often expressed directly [77]. In this study, there were 41% (80/195) Asian participants and 34.4% (67/195) New Zealand Europeans.

In contrast, in North American White culture, high arousal positive affect (eg, excitement) is the most socially desirable to express [77]. As a result, culture may affect how a digital human’s emotional expressiveness is perceived by people and, subsequently, the effect it has on loneliness, social, and physiological outcomes. It is possible that emotional expressiveness in a digital human may need to be tailored to the culture of its intended users alongside their gender. The results may have been different if the study was conducted in the United States.

### Limitations

This study had several limitations that could affect the generalizability of the results. First, the digital human conversation engine experienced occasional errors with utterance detection and speech-to-text translation during its interactions with people. These errors manifested as behaviors such as interrupting people or asking people to repeat themselves, which could have increased negative mood in some participants [68]. However, the errors were not more or less frequent in any of the experimental conditions.

Another possible limitation is that the study was conducted in a community sample predominantly comprised of young adult students. It is unclear whether the findings would apply to other groups who might benefit from a social support intervention with a digital human, such as older adults (who could have lower digital literacy and more challenges with using a digital human program) or particular patient samples (who may be considerably more stressed at baseline). In addition, this study focused on gender differences between men and women; therefore, it is unknown how gender-diverse persons might respond to emotional expressiveness in a digital human, including the effects on their loneliness, social, and physiological outcomes.

Another consideration is that other features of the digital human could have inadvertently influenced people’s responses to it. For example, research has shown that the proportion of a computer agent’s facial features may influence personality perceptions [78]. A narrower face and smaller eyes have been associated with perceptions of greater aggression in computer agents [78]. Both narrow and wide faces have been associated with lower perceptions of trustworthiness than a standard width face, and smaller eyes have been rated as less trustworthy than medium-sized eyes in a computer agent [78]. The face design of the digital human in this study was consistent across the neutral and emotional face conditions; however, each of these components of the digital human’s face could have contributed in part to how participants evaluated the digital human. It is also possible that perceived attractiveness and eeriness of the digital human (ie, the uncanny valley effect [79]), as well as prior experiences with digital humans could have influenced the responses. However, because participants were randomized into groups, these factors should not confound the results.

An important consideration is that demographic matching (eg, gender and ethnicity) between the participant and the digital human could have influenced the social responses. The digital human in this study was female, and different results may have been found if a male digital human had been used. In prior research, gender matching has been shown to influence preferences toward computer agents [80], and ethnicity matching has increased perceptions of trustworthiness [81], social presence [82], and credibility toward computer agents [83]. Ethnicity matching has also been shown to increase closeness, disclosure comfort, and future relationship expectations with computer agents [84].

In this study, the digital human was of mixed ethnicity (New Zealand Māori, New Zealand European, Indian, and European), and the participants were predominantly New Zealand European or Asian, but all resided in New Zealand. There was no significant difference between groups in the proportions of participants of each ethnicity; therefore, this would not have affected our main results. Nevertheless, across the entire sample, Asian ethnicity was related to greater perceived closeness with the digital human (*P*=.01). This may be because of the tendency for Asian cultures to be more accepting of robotic technologies in general [85]. Matching the user’s accent and conversational style has also been shown to increase acceptability and trust with robots and computer agents [86-90]. In this study, the digital human spoke in a local New Zealand accent; however, she did not match the conversational style of participants. We do not have data on participants’ accents from this study.

The effects of demographic and conversational style matching may be because of the similarity-attraction hypothesis, which predicts that people like others more who are more similar to themselves [91]. This social phenomenon has been shown to apply to relationships with computers [46]. Matching the digital human’s demographic characteristics to each participant was not feasible in this study; however, future research could investigate how this might affect the results. The similarity-attraction hypothesis may also explain some gender effects, as women are generally more emotionally expressive than men.

Finally, the expressions in the emotional face condition may not have been expressive enough to elicit changes in outcomes for users. It is possible that more pronounced emotional expressions may have resulted in different findings. In addition, humanlike behaviors performed by the neutral face condition could have been interpreted as showing interest, which may have influenced the results (eg, head movements while listening and eyebrow raises while speaking).

### Future Research

The results of this study indicate several important areas for future research. First, experimental research is needed to investigate the generalizability of the results at the intersection of gender and other demographic or health characteristics (eg, ethnicity, age, and disability). In addition, this study did not examine the effect of emotional expressiveness in a digital human on individuals who are gender diverse, and this topic should be explored in future research.

Second, it is possible that the results may have been affected by the degree of emotional expression in the digital human’s face and the types of emotions that the digital human expressed during the interaction (ie, a mixture of self- and other focused emotions and positive and negative emotions). The expression of these types of emotions has been shown to influence perceptions of computer agents and robots in prior research [55,75]. Future studies could investigate whether expressing more pronounced emotions on the face improves outcomes and evaluate the effect of other focused emotional expression in a digital human (eg, compassion and concern) on loneliness, social, and physiological outcomes. It is likely that this style of emotional expression would better mimic a therapeutic or supportive interaction and may result in greater improvements in outcomes than a digital human that expresses mixed or self-focused emotion. Research could also compare the effects of expressing positive, negative, and a mixture of these expressions.

This study identified emotional expressiveness as an important design feature that should be included in female digital human support persons for women. However, there are likely many other digital human behaviors that could contribute to reductions in loneliness, social, and physiological outcomes for people [40]. This study found that men experienced better outcomes with a female digital human that had less emotional expressiveness in the voice and had no face. However, this finding should be further investigated as men (and women) could have responded differently to a male digital human.

### Conclusions

Overall, emotional expressiveness in a female digital human’s voice was associated with perceiving her as more caring and with experiencing greater physiological arousal during a self-disclosure conversation. However, emotion in the voice was associated with lower feelings of support after the interaction. Gender was an important moderator of these effects. The findings indicate that when designing a female digital human support person for women, an emotional voice and the presence of a face may be important features to include. In contrast, when designing a female digital support person for men, a neutral voice and no face may result in better outcomes than a female with emotional expressiveness. The results provide support for the CASA paradigm and may inform the design of computer agent support persons.

## Appendix

Multimedia Appendix 1 CONSORT e-HEALTH checklist (V 1.6.1).

## Abbreviations

ANOVA: Analysis of Variance
CASA: Computers Are Social Actors
CONSORT: Consolidated Standards of Reporting Trials
RCIT: Relationship Closeness Induction Task
